# Coding-complete genome sequences of two hepatitis C virus genotype 5 variants including one assigned to putative subtype 5b

**DOI:** 10.1128/mra.00276-25

**Published:** 2025-09-17

**Authors:** Donald G. Murphy, Annabelle Mouammine, Lisa Lin, Xiaojie Hu, Michael Carpenter

**Affiliations:** 1 Laboratoire de Santé Publique du Québec, Institut National de Santé Publique du Québec 54470, Sainte-Anne-de-Bellevue, Quebec, Canada; 2 National Microbiology Laboratory Branch, Public Health Agency of Canada 41687, Winnipeg, Manitoba, Canada; Katholieke Universiteit Leuven, Leuven, Belgium

**Keywords:** hepacivirus/genetics, hepacivirus/classification, genotype, next-generation sequencing, Quebec, Canada

## Abstract

Hepatitis C virus (HCV) genotype 5 is not known to be genetically diverse, comprising only one subtype. Here, we report the coding-complete genome sequences of two HCV genotype 5 variants, including one classified to the putative subtype 5b, in samples submitted for routine patient management in Quebec, Canada.

## ANNOUNCEMENT

Hepatitis C virus (HCV), belonging to the *Hepacivirus* genus within the *Flaviviridae* family, is a significant cause of liver disease and public health concern. Sequencing of HCV is essential for comprehending viral evolution and classification, molecular epidemiology, public health management, and treatment responses. There are eight genotypes and over 100 subtypes with genotypes differing by 30% to 35% and subtypes by 15% to 25% at the nucleotide level (https://ictv.global/sg_wiki/flaviviridae/hepacivirus, assessed June 2025). Genotype 5, identified in the early 1990s, has one reported subtype (1, 2), with one strain (BF16) not classified under subtype 5a and one recombinant form 2/5 (3, 4).

NS5B sequence analysis (5) of pre-treatment blood samples from Quebec identified four genotype 5 variants, QC510, QC439, QC757, and QC1137, which are not of subtype 5a. The last three showed 86% to 94% nucleotide identity in C/E1 and 92% to 98% in NS5B. QC510, QC757, and QC1137 were collected from West African migrants in 2010, 2014, and 2022, respectively, while QC439 was from a Canadian-born individual sampled in 2009. We report the coding-complete sequence for QC510 and QC757. This research was conducted in accordance with the Declaration of Helsinki (6).

Plasma viral RNA was extracted using the NucliSENS easyMag (bioMérieux) and treated with DNase. cDNA was generated using a locked oligo d(A) cDNA primer, a template-switching oligonucleotide (TSO), and Superscript IV reverse transcriptase (Thermo Fisher). cDNA products were amplified using GXL polymerase (Takara Bio) and cleaned with AMPure XP magnetic beads (Beckman Coulter). Unique 10 bp dual indexes were added using a Nextera XT DNA preparation kit (Illumina) with 2 × 300 bp reads sequenced on an Illumina HiSeq 2000. Data from 600,000 reads were merged, trimmed of the dual indexes and TSO sequences, and filtered for Phred quality >Q30 using BBDuk v 1.0 (Biomatters). The 5′ end was delineated using the longest TSO reads. Reads were mapped using Geneious Assembler v 2022.2.3 (Dotmatics) against a set of 238 HCV genomes (https://ictv.global/sg_wiki/flaviviridae/hepacivirus, assessed June 2023) with the consensus sequence serving as the reference for a second iteration of mapping. Default parameters were applied for all bioinformatic tools.

**Fig 1:**
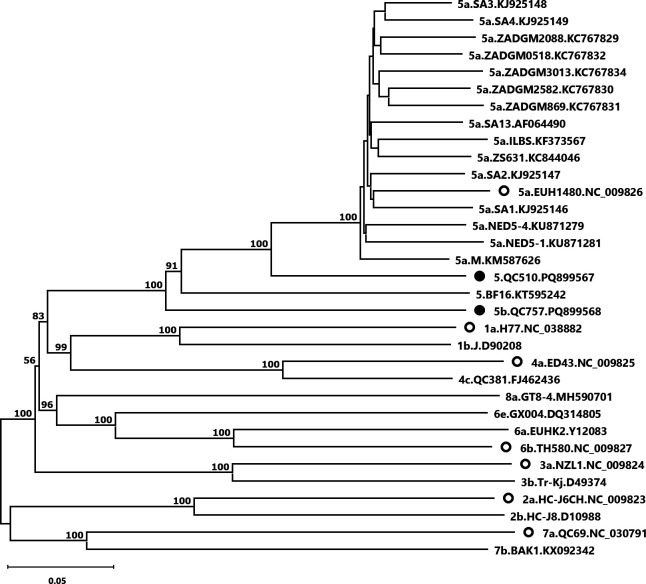
Phylogenetic tree of the complete-coding or nearly complete-coding genome sequences of HCV, including genotype 5 strains QC757 and QC510 identified in this study. HCV sequences of 17 genotype 5 strains and representatives from all other genotypes were retrieved from GenBank. A neighbor-joining tree was built using MEGA11 with the maximum composite likelihood model and default parameters after aligning the sequences using MUSCLE (9). Bootstrap values (based on 1,000 iterations) are shown at each node, except within subtype 5a. Branch labels indicate the HCV subtype (when applicable), strain name, and GenBank accession number. QC757 and QC510 are represented by a black circle, while GenBank HCV reference sequences are indicated by an open circle. The scale bar represents nucleotide substitutions per site.

The open reading frames of QC510 and QC757 comprised 9,048 bases, including the stop codon. The complete 5′ untranslated region (UTR) sequence was 340 bases for QC510 and 341 bases for QC757. The 3′ UTR was complete up to the poly (U/UC) tract. Both genomes had a G + C content of 57% and a mean sequencing depth of ~250. Fig. 1 shows that both fall within genotype 5 but are distantly related to genotype 5a. QC757 exhibited 23% to 24% nucleotide divergence with genotype 5a sequences, while QC510 showed a divergence of 16% to 18% (Fig. 1). No recombination was detected by the Recombination Detection Program - RDP4 (7). QC757, QC439, and QC1137 can be assigned to putative subtype 5b as they meet the criteria for the designation of a new subtype (8). QC510 and BF16, however, cannot currently be assigned to a specific subtype within genotype 5.

This study confirms the genetic diversity of HCV genotype 5 and offers insights into the origin, diversification, and evolutionary history of HCV genotype 5.

## Data Availability

The complete genome sequences of HCV QC757 and QC510 are available in GenBank, under the accession numbers PQ899568 and PQ899567. The raw sequence data are available under BioProject PRJNA1194155 with SRA accession numbers SAMN45171765, SAMN45171766.
